# Early pregnancy fasting plasma glucose and lipid concentrations in pregnancy and association to offspring size: a retrospective cohort study

**DOI:** 10.1186/s12884-016-0846-7

**Published:** 2016-03-17

**Authors:** Bin Liu, Huizhen Geng, Juan Yang, Ying Zhang, Langhui Deng, Weiqing Chen, Zilian Wang

**Affiliations:** Department of Obstetrics and Gynecology, The First Affiliated Hospital of Sun Yat-sen University, 58 Zhongshan Road II, Guangzhou, 510080 PR China; Department of Laboratory Medicine, The First Affiliated Hospital of Sun Yat-sen University, Guangzhou, 510080 PR China; Department of Medical Statistics and Epidemiology, School of Public Health, Sun Yat-sen University, Guangzhou, 510080 China

**Keywords:** Fasting plasma glucose, Triglyceride, Gestational diabetes mellitus, Fetal growth, Lipid metabolism

## Abstract

**Background:**

Hyperlipidemia and high fasting plasma glucose levels at the first prenatal visit (First Visit FPG) are both related to gestational diabetes mellitus, maternal obesity/overweight and fetal overgrowth. The purpose of the present study is to investigate the correlation between First Visit FPG and lipid concentrations, and their potential association with offspring size at delivery.

**Materials and methods:**

Pregnant women that received regular prenatal care and delivered in our center in 2013 were recruited for the study. Fasting plasma glucose levels were tested at the first prenatal visit (First Visit FPG) and prior to delivery (Before Delivery FPG). HbA1c and lipid profiles were examined at the time of OGTT test. Maternal and neonatal clinical data were collected for analysis. Data was analyzed by independent sample *t* test, Pearson correlation, and Chi-square test, followed by partial correlation and multiple linear regression analyses to confirm association. Statistical significance level was *α* =0.05.

**Results:**

Analyses were based on 1546 mother-baby pairs. First Visit FPG was not correlated with any lipid parameters after adjusting for maternal pregravid BMI, maternal age and gestational age at First Visit FPG. HbA1c was positively correlated with triglyceride and Apolipoprotein B in the whole cohort and in the NGT group after adjusting for maternal age and maternal BMI at OGTT test. Multiple linear regression analyses showed neonatal birth weight, head circumference and shoulder circumference were all associated with First Visit FPG and triglyceride levels.

**Conclusion:**

Fasting plasma glucose at first prenatal visit is not associated with lipid concentrations in mid-pregnancy, but may influence fetal growth together with triglyceride concentration.

## Background

The intrauterine metabolic environment is of great importance for the development of offspring [1]. Gestational diabetes mellitus (GDM) is a common metabolic complication that may result in an adverse intrauterine environment and lead to fetal overgrowth [2–4], as well as short- and long-term complications for the offspring [5–8].

Hyperlipidemia, a disorder that may occur in GDM mothers [9] also contributes to the adverse intrauterine environment [10], and often occurs along with hyperglycemia [11]. Early detection of hyperlipidemia in GDM patients is of great importance. A previous study found that fasting plasma glucose (FPG) at the first prenatal visit is related to glycemic metabolism in the mid-gestational period (24-28 weeks) [12]. However, whether FPG at the first prenatal visit is associated with mid-gestational lipid metabolism has not been reported.

Recently, we reported that maternal pre-gravid BMI may influence FPG levels, and FPG at the first prenatal visit is associated with fetal growth [13]. Since maternal obesity/overweight is often complicated with hyperlipidemia [14], and hyperlipidemia may cause of fetal overgrowth [10], we are interested in studying any possible relationship between maternal FPG, lipid metabolism and fetal growth.

In this respect, the primary purpose of the present study is to investigate the relationship between FPG at the first prenatal visit and lipid concentration in the mid-gestational stage. We also aim to examine the association of maternal FPG and lipid metabolism with neonatal birth size.

## Methods

### Study population

Singleton pregnant women who underwent a FPG test at the first prenatal visit (between 10 and 24 gestational weeks), received regular prenatal care (including a lipid concentration test at the time of OGTT test), and delivered in our center from January to December 2013 were recruited for the present study.

The diagnosis of GDM is based on a 75-g Oral Glucose Tolerance Test (OGTT) performed at 24-28 gestational weeks, according to the ADA criteria (fasting ≥ 5.1 mmol/L, 1 h ≥ 10.0 mmol/L, 2 h ≥ 8.5 mmol/L). Pregnant women with overt DM before pregnancy or treated with insulin during gestation were excluded in the present study. All pregnant women complicated with GDM were treated with diet.

The study has been approved by the ethical committees of the The First Affiliated Hospital of Sun Yat-sen University [Application ID: (2014)093] , and all participants provided written informed consent.

### Clinical data collection

At the first prenatal visit, maternal age, parity, the first day of the woman’s last menstrual period (LMP) and weight before pregnancy were self-reported, and maternal height was measured with a fixed stadiometer. Maternal weights at the first prenatal visit, at the time of OGTT test, and just prior to delivery were measured with a calibrated digital scale and recorded for analysis.

Gestational age was calculated from the first day of the woman’s last menstrual period (LMP) and further confirmed by early obstetric ultrasound. If the gestational age calculated from the LMP was different from that calculated by the early ultrasound, the gestational age from the early ultrasound was used [15]. In the case of in vitro fertilization, the gestational age was calculated using oocyte retrieval or co-incubation date and adding 14 days [16].

Measured neonatal parameters included birth weight, birth length, head circumference, shoulder circumference, and birthing method (vaginal vs. cesarean). Immediately following birth, the birth weight was measured with a calibrated electronic scale, birth length was measured with an infantometer, and head and shoulder circumferences were measured with a nylon tape.

### Laboratory measurements

Fasting plasma glucose levels were measured using venous plasma obtained after at least 8 hours of fasting during the first prenatal visit and the morning following administration for delivery. A 75-g Oral Glucose Tolerance Test (OGTT) was performed between 24 and 28 gestational weeks. At the same time, maternal blood samples were also obtained for the examination of HbA1c and lipid profiles including triglyceride, cholesterol, low density lipoprotein, high density lipoprotein, apolipoprotein A1, apolipoprotein B, apolipoprotein E, and lipoprotein-a.

Plasma glucose levels were measured using a GODPAP kit (Human, Wiesbaden, Germany). Cholesterol and triglyceride (TG) levels were measured using an enzymatic colorimetric test kit (Human, Wiesbaden, Germany). High-density lipoprotein (HDL) and low-density lipoprotein (LDL) levels were determined by homogeneous assay using liquicolor kits (Human, Wiesbaden, Germany). Apolipoprotein A1 and Apolipoprotein B were measured with standard enzymatic assay using ApoA and ApoB kits (Human, Beckman, USA). Apolipoprotein E was examined by an immunoturbidimetry assay using APO E AUTO kit (Human, Sekisui, Japan). Lipoprotein-a was examined by a particle-enhanced turbidimetric immunoassay (PETIA) kit (Human, Diagnostic Systems, China). HbA1c was measured by high-performance liquid chromatography (HPLC) using a VARIANT II TURBO HbA1c Kit (Human, Bio-Rad, CA, USA).

### Statistical analysis

Data was analyzed using SPSS Version 17.0. Continuous and normal distributed variables were described as mean ± SD and analyzed by independent sample *t* test or Pearson correlation. Categorical variables were described as proportions and examined by Chi-square test. Partial correlations were applied to describe the relationship between maternal glucose and lipid parameters in the whole cohort, GDM and NGT groups. The false discovery rate was controlled by the Benjamini and Hochberg Method [17]. Multiple linear regression analyses were conducted to study the association between maternal glucose/lipid parameters and neonatal birth size. A p-value of 0.05 or less was considered significant.

## Results

### Baseline characteristics of the study population

1546 pregnant women were recruited for this study and in this cohort, eighteen pregnant women were obese (pregravid BMI > =30 kg/m^2^), 174 were overweight (pregravid BMI:25-29.9 kg/m^2^), 1261 were in normal range BMI (pregravid BMI:18.5-24.9 kg/m^2^) and 90 were lean (pregravid BMI < 18.5 kg/m^2^). Of these women, 276 developed gestational diabetes mellitus (GDM) and 1270 were of normal glucose tolerance (NGT) during pregnancy. Pregnant women in the GDM group were older, contained more nullipara, had higher BMI prior to pregnancy, at the first prenatal visit and at the time of OGTT test compared to the NGT group, but pre-partum BMI between the groups were similar (Table 1).

**Table 1:** Baseline characteristics of the research population

	GDM	NGT	P
Number	276	1270	
Age (mean ± SD)	31.85±4.24	29.42±3.82	<0.001
Parity (%)			0.002
Nullipara	234(84.7)	969(76.2)	
Multipara	42(15.2)	301(23.7)	
Maternal BMI (kg/m^2^)			
Before pregnancy	21.20±3.00	20.47±2.60	<0.001
At first prenatal visit	22.60±3.26	21.63±2.80	<0.001
At OGTT test	24.46±3.09	23.65±2.65	<0.001
At delivery	25.76±3.16	26.04±2.87	0.195
Fasting plasma glucose (mmol/L)			
First Visit FPG	4.46±0.51	4.27±0.38	<0.001
OGTT FPG	4.61±0.53	4.27±0.31	<0.001
Before Delivery FPG	5.22±1.29	4.90±0.99	<0.001
HbA1c (%)	5.04±0.42	4.82±0.31	<0.001
HbA1c (mmol/mol)	31.83±4.63	29.37±3.42	<0.001
Lipid profile			
Triglyceride (mmol/L)	2.31±0.84	2.09±0.76	<0.001
Cholesterol (mmol/L)	6.09±1.02	6.11±0.99	0.754
Low density lipoprotein (mmol/L)	3.26±0.86	3.30±0.81	0.452
High density lipoprotein (mmol/L)	1.82±0.35	1.85±0.33	0.117
Apolipoprotein A1 (g/L)	2.05±0.31	2.01±0.34	0.103
Apolipoprotein B (g/L)	0.94±0.17	0.94±0.18	0.820
Apolipoprotein E (mg/L)	48.32±13.28	46.66±12.67	0.050
Lipoprotein-a (mg/L)	298.70±300.33	307.35±265.39	0.632
Neonatal gender (%)			0.947
Male	143(51.8)	969(76.2)	
Female	133(48.1)	301(23.7)	
Gestational age (day)	271.33±11.70	273.94±11.91	<0.001
Birth weight (g)	3105.49±496.54	3165.55±460.05	0.063
Birth length (cm)	48.96±2.63	49.33±2.39	0.022
head circumference (cm)	33.27±1.98	33.35±1.73	0.583
shoulder circumference (cm)	34.33±2.52	34.45±2.32	0.482
Birth method (%)			0.549
Vaginal	121(43.8)	584(45.9)	
Cesarean	155(56.1)	686(54)	

Neonates of GDM mothers were born at an earlier gestational age and had a shorter birth length compared to neonates from NGT mothers. Gender of neonates, birth weight, head circumference, shoulder circumference, and route of birthing were similar between groups (Table 1). Similar maternal pre-partum BMI and neonatal birth weight between GDM and NGT groups may be a result of diet therapy for GDM pregnant women.

With respect to glucose metabolism, the GDM group had a higher fasting glucose concentration compared to the NGT group at the first prenatal visit (First Visit FPG), at OGTT test (OGTT FPG), and prior to delivery (the morning or next morning of administration for delivery, Before Delivery FPG). The GDM group also had higher HbA1c levels than the NGT group at the time of the OGTT test. Regarding lipid metabolism, triglyceride and Apolipoprotein E levels were higher in the GDM group, but no difference was observed when comparing other lipid parameters between the groups (Table 1).

### Correlation between maternal glycemia and lipid profile

The relationship between glycemia and lipid profile was first analyzed using a univariate correlation followed by a partial correlation taking related confounders into consideration. Analyses were completed in the whole cohort as well as the individual GDM and NGT groups.

The partial correlation between FPG concentration at first prenatal visit and lipid profile was adjusted for maternal pregravid BMI, maternal age and gestational age at First Visit FPG. As shown in Table 2, First Visit FPG was not correlated with any lipid parameters in the whole cohort, or in the NGT/GDM groups.

**Table 2:** Partial Correlation between fasting plasma glucose, HbA1c and lipid profile

	First Visit FPG		HbA1c	
	R'	P	R'	P
*Whole research population*				
Triglyceride	0.045	0.275	0.072	0.032*
Cholesterol	0.046	0.275	0.003	0.919
Low density lipoprotein	0.042	0.275	0.012	0.757
High density lipoprotein	-0.010	0.712	-0.026	0.757
Apolipoprotein A1	-0.010	0.712	0.013	0.757
Apolipoprotein B	0.034	0.299	0.071	0.032*
Apolipoprotein E	0.037	0.299	-0.013	0.757
Lipoprotein-a	-0.014	0.712	-0.015	0.757
*GDM group*				
Triglyceride	0.020	0.946	0.028	0.898
Cholesterol	-0.004	0.946	-0.028	0.898
Low density lipoprotein	0.023	0.946	-0.038	0.898
High density lipoprotein	-0.076	0.592	0.022	0.898
Apolipoprotein A1	-0.078	0.592	-0.008	0.898
Apolipoprotein B	0.046	0.916	0.033	0.898
Apolipoprotein E	0.006	0.946	-0.048	0.898
Lipoprotein-a	-0.110	0.592	-0.015	0.898
*NGT group*				
Triglyceride	0.042	0.29	0.076	0.036*
Cholesterol	0.062	0.232	0.016	0.886
Low density lipoprotein	0.049	0.290	0.032	0.558
High density lipoprotein	0.014	0.706	-0.039	0.485
Apolipoprotein A1	0.004	0.884	0.008	0.886
Apolipoprotein B	0.029	0.496	0.085	0.032*
Apolipoprotein E	0.043	0.290	-0.004	0.886
Lipoprotein-a	0.019	0.684	-0.010	0.886

Partial correlations between First Visit FPG and lipid profiles were adjusted for maternal age, pre-gravid BMI and the gestational age at the First Visit FPG. Partial correlations between HbA1c and lipid profiles were adjusted for maternal age, maternal BMI at the time of the OGTT test. All P values were corrected using the BH method (1995)

**Fig. 1:**
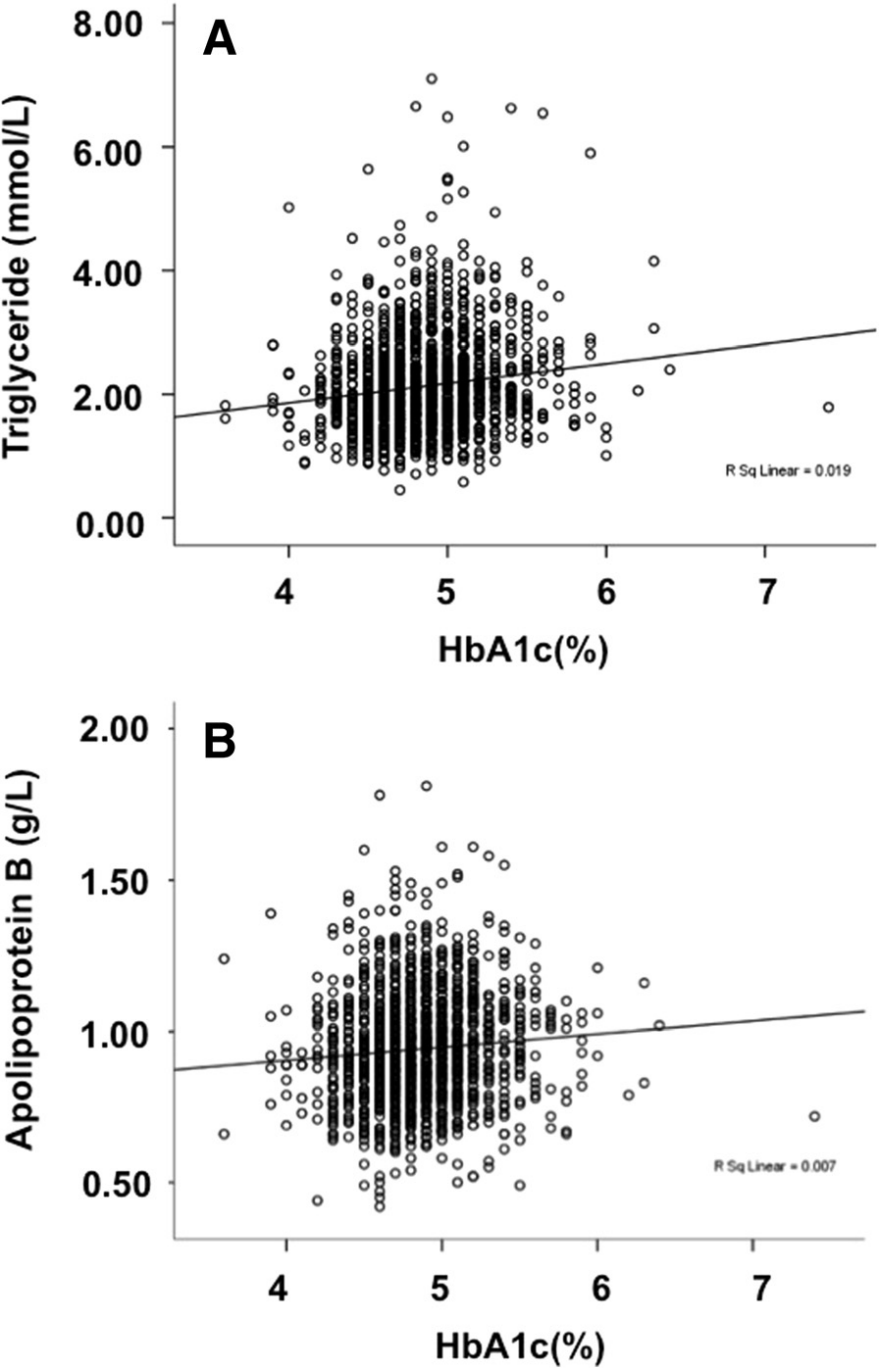
Relationship between HbA1c and lipid concentrations at the time of OGTT test. **a**) Correlation between HbA1c and triglyceride concentration at the time of OGTT test; **b**) Correlation between HbA1c and apolipoprotein B concentration at the time of OGTT test

HbA1c was positively correlated with triglyceride levels in the whole cohort (Fig. 1a, partial correlation coefficient *r’* = 0.072, adjusted P = 0.032) and in the NGT group (partial correlation coefficient *r’* = 0.076, adjusted P = 0.032) after adjusting for maternal age and maternal BMI at OGTT test. HbA1c was also correlated with Apolipoprotein B levels in the whole cohort (Fig. 1b, partial correlation coefficient *r’* = 0.071, P = 0.032) and in the NGT group (partial correlation coefficient *r’* = 0.085, P = 0.032). HbA1c was not correlated with any lipid parameters in the GDM group.

### Maternal glucose levels and lipid metabolism impact fetal growth

Fetal growth is influenced by maternal energy metabolic status, and FPG at first prenatal visit is associated with neonatal birth weight. Therefore, we further studied the impact of both maternal glucose and lipid concentration on fetal growth.

We conducted univariate correlations between neonatal birth weight, length, head circumference, shoulder circumference, and maternal glucose and lipid concentrations followed by partial correlations taking gestational age and pregravid BMI as confounders. In the whole cohort, neonatal birth weight, head circumference and shoulder circumference were all positively correlated with First Visit FPG, OGTT FPG, triglyceride and Apolipoprotein E, but not with other glucose or lipid parameters (Table 3). Birth length was not correlated with any glucose or lipid parameters in this research cohort.

**Table 3:** Partial Correlation between maternal glycemic/lipid metabolism and neonatal birth weight, length, head circumference and shoulder circumference

	Birth weight		Birth length		head circumference		shoulder circumference	
	R'	P	R'	P	R'	P	R'	P
*Glucose metabolism*								
First Visit FPG	0.098	<0.001	0.047	0.086	0.071	0.010	0.084	0.002
OGTT FPG	0.094	0.001	0.033	0.232	0.055	0.048	0.091	0.001
OGTT 1hr	0.029	0.287	-0.040	0.148	0.052	0.061	0.052	0.058
OGTT 2hr	0.027	0.327	0.012	0.677	0.035	0.202	0.049	0.075
Before Delivery FPG	0.011	0.702	-0.022	0.430	-0.017	0.549	0.020	0.480
HbA1c	-0.013	0.647	-0.026	0.350	-0.024	0.382	-0.019	0.495
*Lipid metabolism*								
Triglyceride	0.100	<0.001	-0.003	0.909	0.094	0.001	0.120	<0.001
Cholesterol	0.018	0.518	-0.010	0.710	0.006	0.815	0.024	0.378
Low density lipoprotein	-0.005	0.843	-0.018	0.526	-0.015	0.578	-0.001	0.979
High density lipoprotein	-0.011	0.701	0.022	0.433	-0.049	0.074	-0.039	0.159
Apolipoprotein A1	-0.008	0.760	-0.002	0.945	-0.015	0.576	-0.019	0.484
Apolipoprotein B	-0.016	0.564	-0.032	0.252	0.008	0.773	0.028	0.314
Apolipoprotein E	0.062	0.025	-0.007	0.813	0.064	0.020	0.077	0.006
Lipoprotein-a	-0.033	0.235	-0.031	0.262	-0.042	0.129	-0.026	0.342

All partial correlations were adjusted for gestational age and pregravid BMI

Next, we conducted a multiple linear regression analysis to investigate the association between maternal clinical characteristics (BMI, GDM), glucose/lipid parameters and neonatal birth size (birth weight, head circumference and shoulder circumference). The regression analysis showed that neonatal birth weight, head circumference and shoulder circumference were all best described by models that included First Visit FPG, triglyceride, maternal pregravid BMI and gestational age (Table 4).

**Table 4:** The association between maternal glucose/ lipid parameters and neonatal birth size

	Birth weight			Head circumference			Shoulder circumference		
Effect	Estimate beta coefficient	SE	P	Estimate beta coefficient	SE	P	Estimate beta coefficient	SE	P
First Visit FPG	0.073	24.363	0.001	0.080	0.056	0.001	0.090	0.072	<0.001
OGTT FPG	NS			NS			NS		
Triglyceride	0.070	13.238	0.001	0.049	0.102	0.040	0.065	0.131	0.005
Apolipoprotein E	NS			NS			NS		
Pregravid BMI	0.146	3.846	<0.001	0.108	0.016	<0.001	0.132	0.021	<0.001
GDM	NS			NS			NS		
Gestational Age	0.588	0.870	<0.001	0.426	0.004	<0.001	0.481	0.005	<0.001

## Discussion

Fasting plasma glucose (FPG) at the first prenatal visit is related to the diagnosis of GDM [12], maternal pre-gravid BMI and neonatal birth weight [13]. Since hyperlipidemia is an important link between GDM [9], maternal obesity/overweight [18] and fetal overgrowth [10], we explored the relationship between First Visit FPG and lipid concentrations, and their potential influence on fetal growth. The result of the present study showed that, although First Visit FPG was not correlated with any lipid parameters at mid-gestation, it was associated with neonatal birth weight, head circumference and shoulder circumference together with maternal triglyceride levels.

The crosstalk between glucose and lipid metabolism is well established [19]. During pregnancy, hyperglycemia is typically accompanied by hyperlipidemia [20] and together they promote an adverse metabolic intrauterine environment and lead to macrosomia [6]. FPG levels at the first prenatal visit is strongly correlated with glucose tolerance in mid pregnancy [12], but whether FPG levels can influence lipid metabolism is unknown.

In the present study, First Visit FPG was not correlated with any of the measured lipid parameters in mid-gestation. However, HbA1c concentration correlated with triglyceride and Apolipoprotein B levels in the NGT group. Since HbA1c concentration represents the average glucose level 2-3 months before testing [21], mid-pregnancy triglyceride and Apolipoprotein B levels would be related to earlier stage glucose metabolism. However, a single test of FPG at the first prenatal visit was not able to predict hyperlipidemia at the mid-gestational stage.

Fetal growth is influenced by maternal glucose levels [22] and lipid metabolism [23], so in the present study, we analyzed glucose and lipid factors on neonatal growth parameters. We found that maternal First Visit FPG and triglyceride levels were correlated with neonatal birth weight, head circumference and shoulder circumference, after adjusting for gestational age and GDM. Other lipid parameters, HbA1c and after meal glucose (OGTT 1 and 2 hour) were not associated with any neonatal birth size characteristics. This result suggests that maternal fasting glucose and triglyceride concentrations play an important role in fetal growth.

The association between maternal triglyceride and neonatal birth weight has been reported in previous studies [24, 25], and our results support these studies in a much larger study population. Neonatal head circumference has been shown to be negatively correlated with low-density lipoprotein in cord plasma [26]; however, to the best of our knowledge, the positive association between maternal triglyceride and neonatal head and shoulder circumference is first described in the present study. Our recently published article found that First Visit FPG was associated with neonatal birth weight [13], and the present study revealed the association between First Visit FPG levels and neonatal head and shoulder circumference in a new research population.

There are several limitations in the present study. First of all, variation of lipid concentration is considerable during gestation [27], but we only obtained the lipid values at the time of the OGTT test. Additionally, since the BMI of most participants in the present cohort were within the normal range, the result of the present study would be applicable mainly in pregnant women with normal BMI. Further, although the present study has provided insight into the association between glucose, lipid factors and neonatal birth size, the retrospective design could be seen as another limitation. Therefore, further prospective studies measuring lipid concentrations across multiple gestational times in a wide ranging population is needed to increase validation of the result.

## Conclusion

In summary, mid-pregnancy triglyceride levels are correlated with earlier stage glucose metabolism, but a single FPG test is not sufficient to predict triglyceride concentrations in the mid-gestational period. In addition, neonatal birth weight, head circumference, and shoulder circumference were all associated with maternal fasting glucose levels at the first prenatal visit and triglyceride levels at mid-gestation, but not with after-meal glucose levels at the time of OGTT test or with other lipid parameters.
